# Maximizing gerrymandering through ising model optimization

**DOI:** 10.1038/s41598-021-03050-z

**Published:** 2021-12-08

**Authors:** Yasuharu Okamoto

**Affiliations:** 1 System Platform Research Laboratories, NEC Corporation, 1753 Shimonumabe, Nakahara-ku, Kawasaki, Kanagawa 211-8666 Japan; 2 NEC-AIST Quantum Technology Cooperative Research Laboratories, 1-1-1 Umezono, Tsukuba, Ibaraki 305-8568 Japan

**Keywords:** Applied mathematics, Computational science

## Abstract

By using the Ising model formulation for combinatorial optimization with 0–1 binary variables, we investigated the extent to which partisan gerrymandering is possible from a random but even distribution of supporters. Assuming that an electoral district consists of square subareas and that each subarea shares at least one edge with other subareas in the district, it was possible to find the most tilted assignment of seats in most cases. However, in cases where supporters' distribution included many enclaves, the maximum tilted assignment was usually found to fail. We also discussed the proposed algorithm is applicable to other fields such as the redistribution of delivery destinations.

## Introduction

Gerrymandering is a generic term for methods that rig electoral districts assuming advantage to a specific party. As long as geographical districts are adopted, the demarcation of districts is inevitable. Electoral districts are also a unit of voters artificially defined by electoral legislation. If this artificial demarcation is intentional, the neutrality of being a geographical division will disappear, and the problem of partisan gerrymandering arises. In terms of its practical effect on the exercise of voting rights, gerrymandering corresponds to systematic control of wasted votes.

There are two techniques widely known in gerrymandering: cracking and packing^1^. The former means the cracking of the opposing party's voters' concentration not to form a majority of the district. On the other hand, the latter is to pack as many of the opposing party’s voters as possible into the same district when it is unavoidable to form a district with most of the opposition. Packing will dilute the number of opponents who may have formed a majority in other districts. Thus, cracking and packing is practically equivalent to a combination of a narrow victory and a big defeat. Cracking and packing make it possible to minimize the number of wasted votes for governing party while maximizing it for the opposition. A voter who is assigned a wasted vote by gerrymandering effectively loses the opportunity to elect a representative. In other words, such a voter would enjoy only the right to cast a wasted vote, which leads to the danger of distorting the normative imperatives established in our time to reflect the people's diverse will.

Gerrymandering has been studied through jurisprudential or sociological approaches since it occurred in the early nineteenth century^2–5^, however, it has also been studied in mathematical science in recent years^6–9^. Studies based on Markov chain Monte Carlo help evaluate districting's intentionality, which would be necessary for gerrymandering certification in court. Puppe and Tasnádi proved that determining whether given geography admits an unbiased districting is an NP-complete problem^9^. Furthermore, in recent years, with the development of information and communication technology, information gerrymandering^10^ and digital gerrymandering^11^ have aroused concern. Although there is no clear definition of digital gerrymandering, it suggests that voting behavior is biased through social networks^12^ and public opinion manipulation by extensive data analysis. Thus, the concept of gerrymandering is expanding in recent times, and it becomes a research area that requires closer integration of social and mathematical science. Such mathematical studies on gerrymandering are still in the early stages. Therefore, various approaches from different disciplines are important and worth considering to shed new light on it.

The combinatorial optimization based on the Ising model employed in this study means the approach where the objective function is formulated by the Ising model with 0–1 binary variables and optimized through metaheuristics such as simulated annealing or tabu search^13^. The approach attains considerable attention recently in terms of the development of quantum annealing^14^. Furthermore, with the progress of quantum annealing, it is noteworthy that dedicated computers^15^, and algorithms for Ising models are appearing in classical computation^16,17^. Consequently, the combinatorial optimization based on the Ising model begins to be applied to various fields and problems such as logistics (delivery route planning)^18^, factories (automated guided vehicle operation)^19^, services (nurse scheduling)^20^, finance (risk management of financial assets)^21^, materials science (metamaterials design)^22^, and drug design (molecular similarity search)^23^.

In this paper, by using combinatorial optimization based on the Ising model^13^, we investigated the extent to which partisan gerrymandering under a two-party system is possible to tilt seats in favor of one party starting from a random but even distribution of supporters for both parties. Our model consists of 70 cells that express the distribution of supporters. Supporters of either Party A or B govern each cell. All districts consist of 5 cells, and the total number of seats in the model is 14. If there is support for three or more cells out of 5 cells in a district for either party, the party will get the seat. We observed that both parties could assign the theoretically maximum number of seats by gerrymandering in 8 out of 10 cases of random distribution of the equivalent supporters when a rook constraint forbade enclaves in a district.

## Methods of computation

### Model of single-seat district

**Figure 1:**
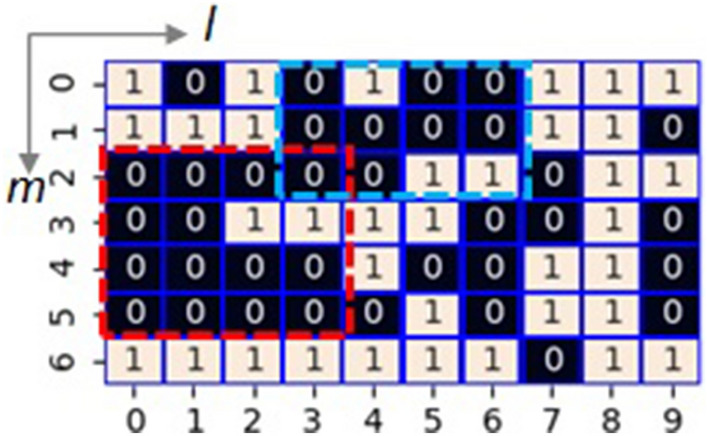
A model of electoral districts with 70 cells arranged in a 7 × 10 rectangle (Case 1) where 1(0) means a cell that Party A(B) has an advantage over B(A). Party A controls 35 cells, whereas Party B controls the remaining 35 cells. Red or blue dotted rectangle designates the areas where Party B has a significant advantage.

The model examined in this study contains *N*_g_ single-seat districts, and each district consists of *N*_c_ cells. Therefore, the total number of cells in the model is *N*_g_ × *N*_c_ (≡ *N*_s_). As shown in Fig. 1, we arrange the *N*_s_ cells in a rectangle of length *m* and width *l*. The party with a majority of the *N*_c_ cells that make up a district gets the seat. Both parties control half of the *N*_s_ cells. Therefore, the powers of both parties are in equilibrium as a whole. Besides, we assume that *N*_s_ is even, and *N*_c_ is odd to balance the two parties' power and decide which wins in each district. We randomly give the cells' position where Party A governs in the (m × l) rectangle as the initial value. We set *N*_g_, *N*_c_, *m*, and *l* as 14, 5, 7, and 10, respectively, unless otherwise stated.

If we can rig the redistricting completely freely in favor of one party from both parties' even distribution, the number of seats won by the party will be $\left\lfloor {\frac{{N_{s} /2}}{\lceil{N_{c} /2}\rceil}} \right\rfloor \left( { \equiv N_{{max}} } \right)$ at the maximum, where $\left\lceil x \right\rceil$ denotes the ceiling function and means the smallest integer greater than or equal to x. Similarly, $\left\lfloor x \right\rfloor$ denotes the floor function and means the largest integer less than or equal to x.

However, we should note that redistricting is usually not unconditional. Thus, we impose a condition to forbid the occurrence of the enclave, called a rook constraint. The rook constraint means that cells in the same district share at least one edge with other cells in the district. In the following, we will explain by assuming that Party A enjoys an advantage over Party B.

### Constrains for districts and cells in the Ising model

We give the constraints that must be satisfied by districts and cells as follows,

$${H}_{cst}^{1}=\sum_{g=0}^{{N}_{g}-1}{\left(\sum_{s=0}^{{N}_{s}-1}{x}_{s}^{g}-{N}_{c}\right)}^{2}+\sum_{s=0}^{{N}_{s}-1}{\left(\sum_{g=0}^{{N}_{g}-1}{x}_{s}^{g}-1\right)}^{2}$$

where ${x}_{s}^{g}$ represents 0/1 binary variables, if we assign cell *s* (*s* = 0, 1…, *N*_s_-1) to district *g*, then ${x}_{s}^{g}$ is one. Otherwise, it is zero. The first term in RHS means the constraint that the number of cells comprising each district is *N*_c_. The second term in RHS means the constraint that each cell belongs to only one district. If these constraints fail, the energy of the objective function will increase as a penalty.

### Counting the number of governing cells in each district

Assuming that *j* is the number of cells in which Party A governs in the district *g*, *j* must satisfy the following constraints.

$${H}_{cst}^{2}=\sum_{g=0}^{{N}_{g}-1}{\left(\sum_{j=0}^{{N}_{c}}{y}_{j}^{g}-1\right)}^{2}+ \sum_{g=0}^{{N}_{g}-1}{\left(\sum_{j=0}^{{N}_{c}}{j\times y}_{j}^{g}-\sum_{s=0}^{{N}_{s}-1}{f(s)\times x}_{s}^{g}\right)}^{2}$$

where ${y}_{j}^{g}$ represents 0/1 ancillary binary variables, if the number of cells where Party A dominates in the district *g* is equal to *j*, then ${y}_{j}^{g}$ is one. Otherwise, it is zero. Besides, $f(s)$ represents that if Party A governs cell *s*, then $f(s)$ is one. Otherwise, it is zero. Note that, unlike other binary variables determined through optimization, we give *f*(*s*) as the input data that reflect Party A or B supporters' distribution. The first term in RHS means that ${y}_{j}^{g}$ is a one-hot vector corresponding to 0 to *N*_c_. The second term in RHS is a constraint that the number of cells in which Party A governs in the district *g* is *j*.

For gerrymandering, we require a driving force to decrease the energy of the objective function during the optimization by redistricting *N*_s_ cells in favor of Party A. We introduce a driving force as follows.

$${H}_{gerr}=\sum_{g=0}^{{N}_{g}-1}\sum_{j=0}^{{N}_{c}}p(j)\times {y}_{j}^{g}$$

where *p*(*j*) acts to increase the number of districts in favor of Party A. Let *j* be the number of cells in each district where party A governs. If $j\le \lceil\frac{{N}_{c}}{2}\rceil-1$, we set $p\left(j\right)>0$ to reduce the districts that are disadvantageous to Party A. On the other hand, if $j\ge \lceil\frac{{N}_{c}}{2}\rceil$, we set $p\left(j\right)<0$ to increase the districts that are advantageous to Party A. After some trial error, we determined *p*(*j*) as follows,

$$p\left( j \right) = \left\{ {\begin{array}{*{20}l} { + \frac{3}{2}} \hfill & {0 \le j \le \lceil\frac{{N_{c} }}{2}\rceil - 1} \hfill \\ { - 2} \hfill & {j = \lceil\frac{{N_{c} }}{2}\rceil} \hfill \\ { - \frac{1}{2}} \hfill & {\lceil\frac{{N_{c} }}{2}\rceil + 1 \le j < N_{c} } \hfill \\ \end{array} } \right.$$

In this setting of *p*(*j*), losing a seat raises the energy whereas gaining a seat lowers the energy, especially when a party just barely wins at $j=\lceil\frac{{N}_{c}}{2}\rceil$ number of votes, which lowers the energy significantly.

Finally, we introduce the rook constraint. Two cells that share an edge led to decreasing the energy of the objective function. We give the rook constraint as follows.

$${H}_{rook}=-\sum_{g=0}^{{N}_{g}-1}\sum_{s, {s}^{^{\prime}}\in {S}_{rook}}{x}_{s}^{g}{x}_{{s}^{^{\prime}}}^{g}$$

Here, *S*_rook_ designates a set in which the two cells *s* and *s*’ share an edge. Thus, the Hamiltonian (*H*_Ising_) of the Ising model is given by

$${H}_{Ising}=A{\times H}_{cst}^{1}+B\times {H}_{cst}^{2}+C\times {H}_{gerr}+D\times {H}_{rook}$$

A, B, C, and D represent hyperparameters that regulate the strength of the constraints and objective function. We determined them by Bayesian optimization (BO) using a Gaussian process^24^. The method is suitable for global optimization of black-box functions with low variable dimensionality but high function evaluation cost, such as the problem treated here. It is also advantageous in that no derivative of the function is required for optimization.

We can find the minimum of *H*_Ising_ by optimizing the Hamiltonian. We used tabu search (TS)^25,26^ to optimize it because TS gave better results than simulated annealing in our preliminary tests. We used Qbsolv for TS; an application program interface supported in D-Wave Ocean (software development kit developed by D-Wave Systems)^27^. Note that Qbsolv is a quantum–classical hybrid solver for quadratic unconstrained optimization problems with binary variables (QUBO), however, in this paper it is simply used as a classical solver that solves QUBO by TS. Although *H*_Ising_ consists of four partial Hamiltonians (${H}_{Ising}=A{\times H}_{cst}^{1}+B\times {H}_{cst}^{2}+C\times {H}_{gerr}+D\times {H}_{rook}$), the division is for the purpose of explaining the formulation, and Qbsolv actually solves one QUBO corresponding to *H*_Ising_, which is the sum of these terms.

Although *H*_rook_ favors cells that share an edge, we observed that this alone did not prevent the district from containing enclaves. If we regard a district as a graph, the absence/presence of enclaves corresponds to the connected/disconnected graph. It is easy to determine whether a graph is connected or not by using graph theory algorithms. We count the number of districts that have enclaves among *N*_g_ districts and use the number (*N*_dis_) as a penalty to the objective function of BO (*H*_BO_) expressed as,

$${H}_{BO}={N}_{cst}^{1}+{N}_{cst}^{2}+{N}_{dis}-{N}_{seat}$$

${N}_{cst}^{1}$ represents the expectation value of ${H}_{cst}^{1}$ by the solution vector *x*_opt_ obtained from optimizing *H*_Ising_ by TS, namely ${N}_{cst}^{1}=\langle {x}_{opt}|{H}_{cst}^{1}|{x}_{opt}\rangle$. If the solution vector satisfies the constraint given by ${H}_{cst}^{1}$, then ${N}_{cst}^{1}$ will be zero. Consequently, this value is an indicator of how much the constraint is not satisfied. The same is true for ${N}_{cst}^{2}=\langle {x}_{opt}|{H}_{cst}^{2}|{x}_{opt}\rangle$. *N*_dis_ also becomes zero if there are no enclaves in all *N*_g_ districts. *N*_seat_ is the number of seats won by Party A, calculated from the solution vector *x*_opt_. Note that *H*_BO_ will be -*N*_max_ if gerrymandering could assign seats completely tilted manner. However, due to the rook constraint, the value may not be reached depending on the supporters’ initial distribution. It is noteworthy that BO takes care of the adjustment of hyperparameters in *H*_Ising_ and the prohibition of enclaves in the districts. We observed that the prohibition of enclaves was difficult to achieve by simply optimizing the Ising model. In this study, we have updated BO a thousand times, and we optimize *H*_Ising_ by TS of 1250 or 5000 trials in each BO update. We carried out BO by using gp_minimize in scikit-optimize^28^. The parameters for gp_minimize was set as n_calls = 1000 (number of function calls), and acq_func = 'EI' (negative expected improvement).

## Results and discussion

### Rook constraint

**Figure 2:**
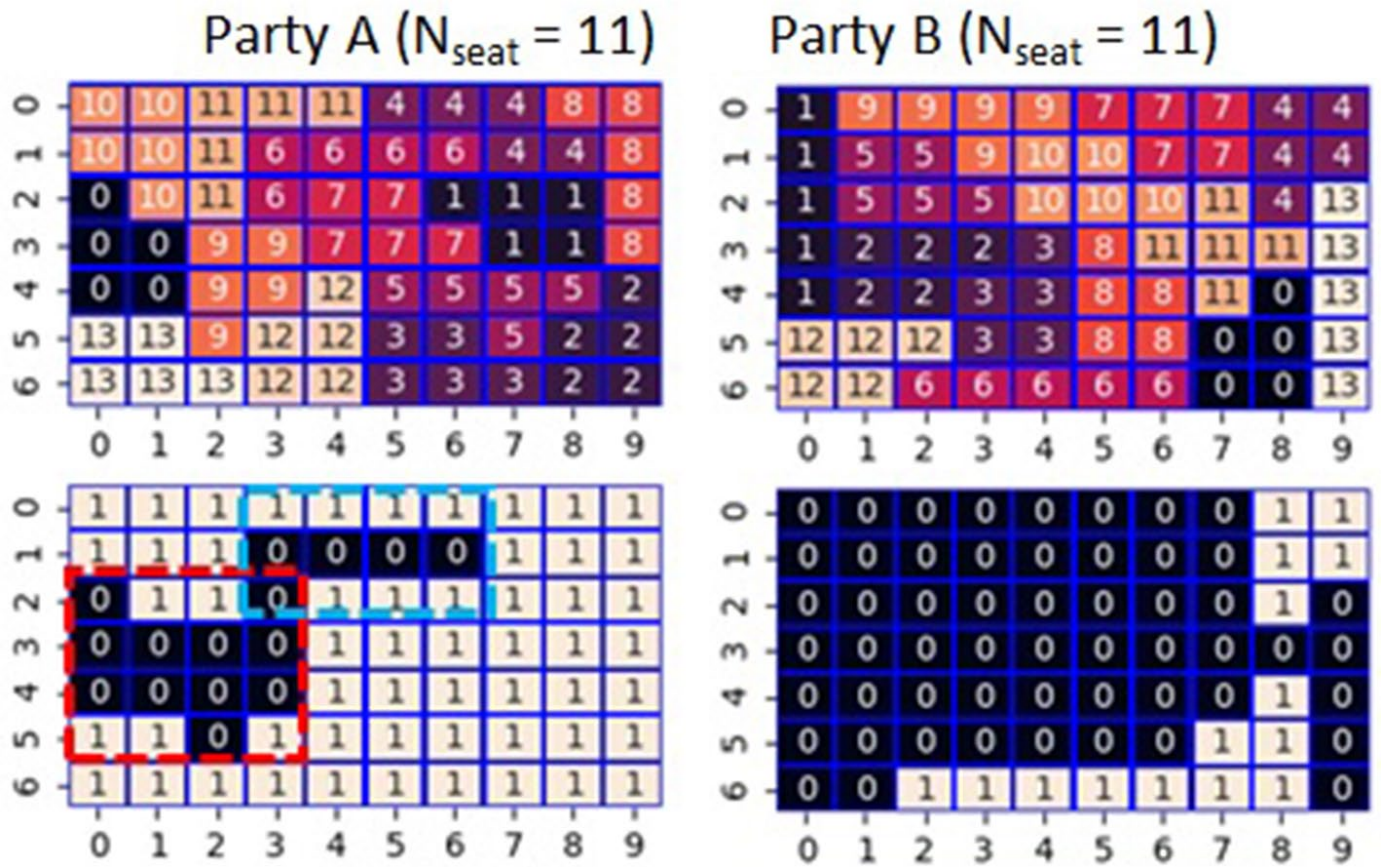
Calculated result of maximizing the number of seats so that party A or B has an advantage with imposing the rook constraint concerning the supporter’s distribution shown in Fig. 1 (Case 1). The top panels show the calculated correspondence between the cells and the 14 districts indexed from 0 to 13. The bottom panels show the assignment of seats based on the calculated districting.

Fig. 1 shows an example of supporters’ distribution. Hereafter, we refer to this example as Case 1. It requires at least 33 governing cells to set the number of assigned seats (*N*_seat_) to *N*_max_(= 11 [in this study]) with 3 to 2 hard-won victories (in other words, the seat is won by one vote). Due to the even distribution in the initial condition, the number of cells where each party governs is 35 among 70 cells; thereby, the allowed number of wasted votes for the governing party is at most two. Only three districts fail to secure seats in *N*_g_ districts. When the number of wasted votes is 2 for the governing party, there are two cases: two districts losing 0 to 5 and one losing 2 to 3, or a combination of one district losing 0 to 5 and two losing 1 to 4. Concerning the distribution of supporters of Case 1 (Fig. 1), under the condition that Party A has an advantage (left side in Fig. 2), it has lost seats in districts 0, 6, and 9. In districts 0 and 6, it lost 0 to 5, whereas, in district 9, it lost 2 to 3. On the other hand, under the condition that Party B has an advantage (right side in Fig. 2), it has lost seats in districts 0, 4, and 6. In districts 0 and 4, it lost 1 to 4, whereas, in district 6, it lost 0 to 5.

It is noteworthy that 87.5% of the cells surrounded by the red dotted lines in Fig. 1 belong to Party B. Packing contributes to Party A by decreasing the own wasted vote and ensuring that Party B's power does not extend outside the area. All three districts where party A was defeated are related to the part surrounded by the red dotted lines. On the other hand, although 75% of the cells surrounded by the blue dotted lines in Fig. 1 belong to Party B, proper cracking of the area is helpful for Party A to win 3 to 2 by controlling the concentration of supporters of Party B. By controlling the wasted vote in this way, the governing party achieves hard-won victories and great defeats, which maximizes the number of seats won. Note that it would be possible that the number of wasted votes for the governing party could be zero or one, but we had not observed such cases in the present study.

**Figure 3:**
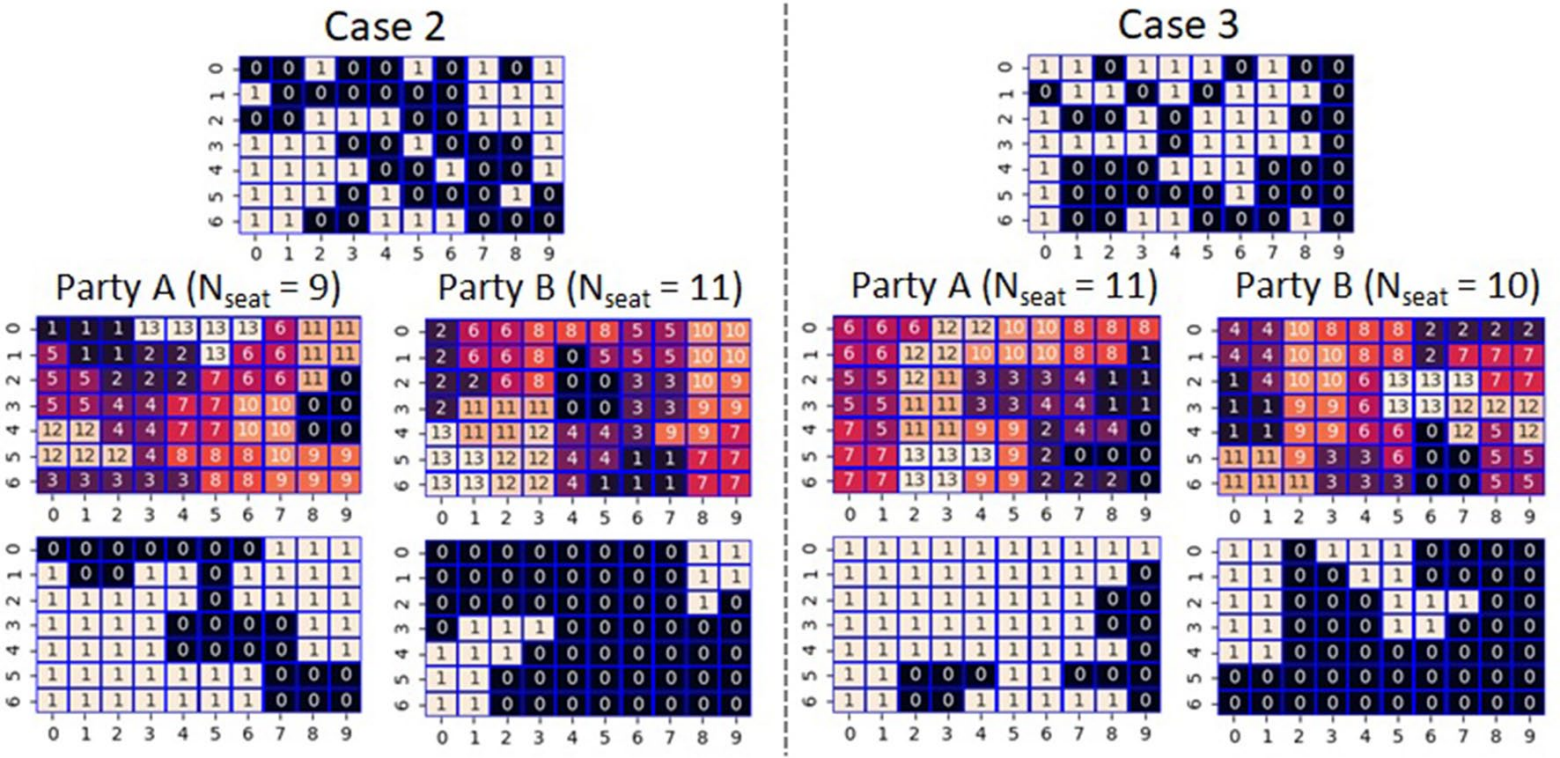
Two cases of gerrymandering with rook constraint. In each case, the top panel shows the distribution of the dominant cells where 1(0) means a cell that Party A(B) has an advantage over B(A). Middle panels show the calculated correspondence between the cells and the 14 districts indexed from 0 to 13. The bottom panels show the assignment of seats based on the calculated districting.

In the above example, the number of seats (*N*_seat_) won by both A and B reached *N*_max_. However, there were cases where the value was less than *N*_max_. Figure 3 shows such examples. In Case 2, the setting that favors Party A results in a *N*_seat_ of only 9. In this case, cells (1,0), (0,2), and (0,5) are enclaves away from the other cells governed by Party A. Similarly, cells (3,5), (4,6), and (5,8) are also enclaves because although they share vertices with other cells governed by Party A, none of them share the edges needed to satisfy the rook constraint. Comparing these six enclaves with the assigned seats, five cells other than the cell (1,0) result in wasted votes. Due to a large number of wasted votes, the *N*_seat_ of Party A remains at 9. As another example, we show Case 3, where *N*_seat_ remains at 10 in the setting in which Party B has an advantage. It is noteworthy that five cells (1,0), (0,2), (1,3), (1,5), and (0,6) are enclaves, and cells (1,0) and (1,5) become wasted votes. These results suggest that whether or not the number of seats won reaches *N*_max_ depends on the number of enclaving cells for a governing party. It appears difficult for enclaves to form a majority because there are no own party cells around them. To verify this, we examined seven more cases. We show a figure similar to Fig. 3 as Fig. S1 in Supplementary Information (SI). In a total of 20 optimizations for these 10 cases, the number of times *N*_seat_ = *N*_max_ was 16, whereas the number of times *N*_seat_ < *N*_max_ was 4. The average values of the enclaves were 1.94 (*N*_seat_ = *N*_max_) and 5 (*N*_seat_ < *N*_max_), respectively. However, although it appears to be a rare example, it is noteworthy that *N*_seat_ is possible to reach *N*_max_ even from a distribution with many enclaves as in Case 6 (Party A) of Fig. S1 in SI. Besides, the Ising model-based approach does not guarantee optimality, thereby even in the examples where *N*_seat_ < *N*_max_, the possibility exists that the true optimum is *N*_seat_ = *N*_max_.

In this study, we optimized the Hamiltonian of the Ising model by TS with every BO update, but its accuracy depended on the number of TS trials (*N*_TS_). When *N*_TS_ was 1250, *N*_seat_ reached *N*_max_ in 11 out of 20 BOs. When we increased *N*_TS_ to 5000 for nine distributions that did not reach *N*_max_ with an *N*_TS_ of 1250, *N*_seat_ reached *N*_max_ in five of them. Consequently, *N*_max_ was reached 16 times out of 20 BOs. Table 1 shows the change in the number of feasible solutions obtained during 1000 BO updates by increasing *N*_TS_ from 1250 to 5000. The result indicates that the improvement of the probability of finding a feasible solution by increasing *N*_TS_ led to the improved the entire solution's accuracy, including BO.

**Table 1:** Comparing the number of times we obtained a feasible solution during 1000 BO updates by TSs with 1250 or 5000 trials.

Case	Governing party	*N*_TS_ = 1250			*N*_TS_ = 5000
		1	2	3	
1	B	46(10)	96(10)	30(10)	53(11)
2	A	40(9)	36(9)	27(8)	75(9)
3	B	28(9)	28(10)	13(10)	47(10)
4	B	57(10)	63(10)	64(10)	84(11)
5	A	83(10)	91(9)	93(10)	97(11)
6	A	46(10)	46(10)	51(10)	69(11)
6	B	51(10)	50(10)	49(10)	110(10)
7	A	101(10)	71(10)	90(10)	150(10)
8	A	46(10)	32(10)	42(10)	71(11)

The number in parentheses indicates the number of seats assigned (*N*_seat_). The TS with 1250 trials was run three times with different random number seeds for the initial value.

### Queen constraint

**Figure 4:**
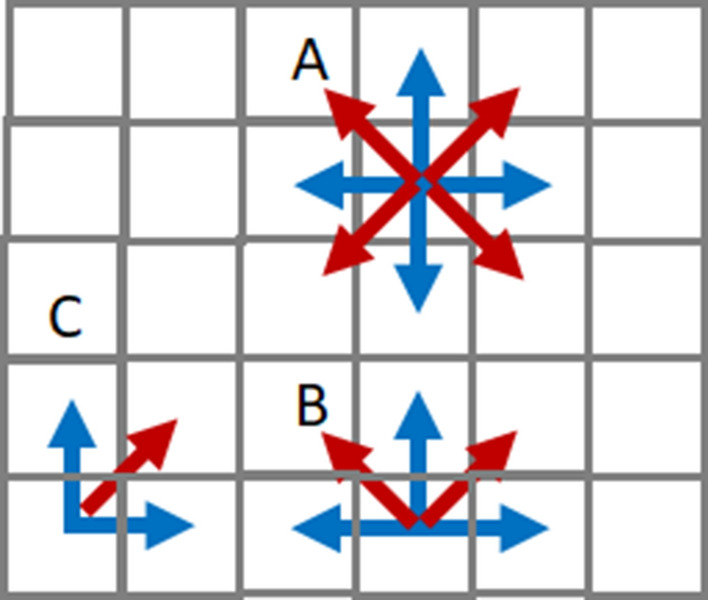
(**A**) The blue and red arrows point to adjacent cells with the rook and queen constraints, respectively. (**B**) At the boundary, the number of adjacent cells under the rook constraint is 3, whereas the number of adjacent cells under the queen constraint is 2. (**C**) At the corner, the number of adjacent cells under the rook constraint is 2, whereas the number of adjacent cells under the queen constraint is 1.

**Figure 5:**
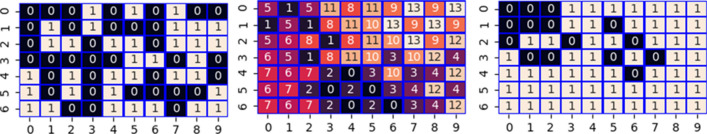
Gerrymandering with queen constraint. The left panel shows the distribution of the supporters where 1(0) means a cell that Party A(B) has an advantage over B(A). Middle panel shows the calculated correspondence between the cells and the 14 districts indexed from 0 to 13. The right panel shows the assignment of seats based on the calculated districting.

In addition to the rook constraint where cells share edges, the queen constraint where cells share vertices is a representative constraint for districting. We observed that the latter constraint makes it more difficult for *N*_seat_ to reach *N*_max_ than the former constraint starting from even distribution. We subsequently considered why the computation with the queen constraint being difficult. As shown (A) in Fig. 4, both the rook and queen constraints have four adjacent cells that satisfy the constraints. However, at the boundary (B) and corner (C), the number of adjacent cells satisfying the constraints is 3 and 2, respectively, in the case of the rook constraint. On the other hand, in the case of the queen constraint, the number of adjacent cells is 2 (at the boundary) and 1 (at the corner). The decrease of the number of adjacent cells at the boundary and the corner in the queen constraint results in an energy disadvantage. Although this is a little ad hoc approach, we changed the energy decrease due to vertex sharing in the partial Hamiltonian of *H*_rook_ at boundaries and corners to twice as much for the queen constraint. As shown in Fig. 5, we observed districts that give the maximum number of seats (*N*_max_) to party A with the queen constraint.

Moreover, although this is a mathematical model that deviates from reality, if we assume that there are periodic boundary conditions on the arrangement of cells (distribution of supporters and districts) to eliminate the effect of boundaries and corners, we can calculate the districts that allow the maximum number of seats (*N*_max_) to one party with the queen constraint starting from even distribution (Fig. S2 in SI).

### Multiple votes per cell

In the simulations so far, the number of votes in each cell (*N*_v_) has been set to one. However, it is easy to extend the formulation to treat the number of votes per cell to *N*_v_(> 1). In this case, the total number of votes in each district changes from *N*_c_ to *N*_c_ × *N*_v_ (≡ *N*_t_). Therefore, in the partial Hamiltonian related to the total number of votes per district, we must change *N*_c_ to *N*_t_. Specifically, we change *N*_c_ to *N*_t_ in *H*^2^_cst_ and *H*_gerr_. This is a trivial change in terms of formulation, but we found that it was quite difficult to get the correct result because of the expansion of search space through the increased total number of votes.

**Figure 6:**
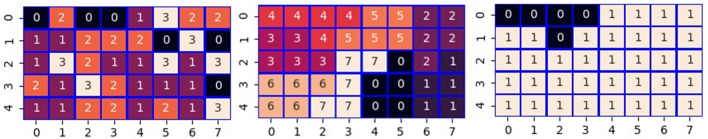
Gerrymandering in a multiple votes case (3 votes/cell) with rook constraint. The left panel shows the number of votes in each cell for Party A. Center panel shows the calculated correspondence between the cells and the eight districts indexed from 0 to 7. The right panel shows the assignment of seats based on the calculated districting.

An example of the calculation is shown below. In a model of eight districts containing a total of 40 cells, each district consists of five cells, and the number of votes per cell is *N*_v_ = 3. The left panel of Fig. 6 shows the number of votes in each cell supporting Party A. The sum of these values in all cells is 60. Since the total number of votes is 120 (= 3 × 40), the number of votes supporting Party B is also 60, and the distribution is even in the initial condition. Since the total number of votes in one district is 15 (= 3 × 5), if either party gets eight or more votes, it will be the winner in that district. Therefore, the maximum number of acquired seats without the constraint of prohibiting enclaves is 7 (= $\lceil\frac{60}{8}\rceil$). The center of Fig. 6 shows the redistricting results obtained from the optimization with rook constraint, and the right panel shows the results of the allocation to Party A and Party B based on the redistricting. We observed Party A has acquired the maximum number of seats that can be allocated.

### Application to other fields

**Figure 7:**
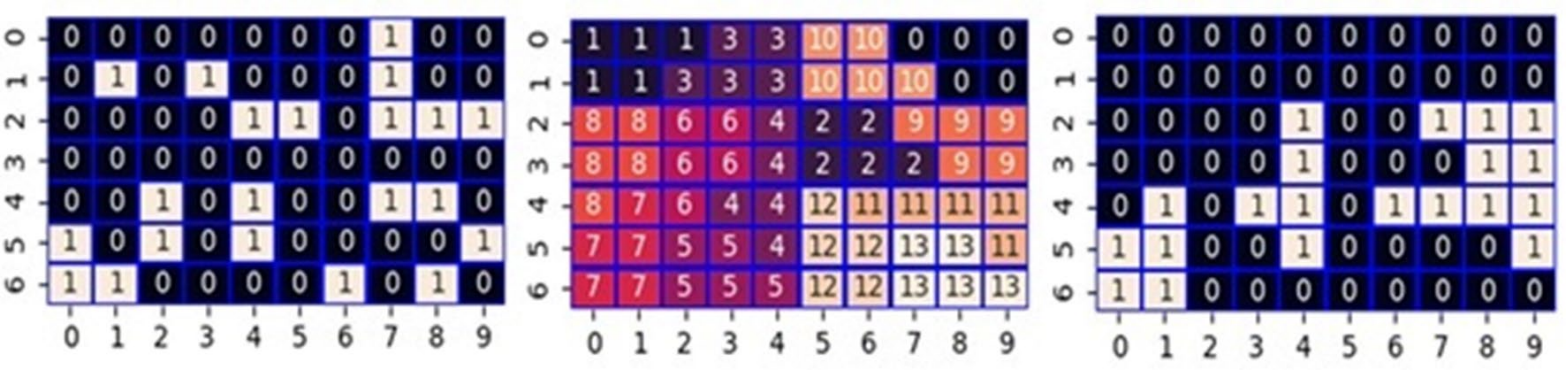
Companies in charge of each delivery destination at preset (left). Calculated correspondence between the delivery destinations and the 14 groups indexed from 0 to 13 (center). The assignment of company to manage new groups (right).

The essence of the algorithm proposed here is to redistribute the disjointed small parts into a compact form according to a set of rules. There appear to be various fields to which such features can be applied. As an example, we will discuss the redistribution of delivery destinations. Company 0 and Company 1 deliver the same product to their customers. Figure 7 (left) shows which company is in charge of each delivery destination at present. Here, the number of delivery destinations handled by company 0 and company 1 is 49 and 21, respectively, so the share ratio is 7:3. The scattered delivery destinations in the figure cause poor efficiency and long transportation distance, increasing carbon dioxide emissions.

Therefore, we group several destinations to form a group, and one of the companies is in charge of all the destinations in a group. The distribution of the group to each company should reflect its current share as much as possible. Within a group, we do not allow enclaves to reduce the transportation distance and increase the delivery efficiency. Besides, we assign each group to the company having more delivery destinations at present. The rule is important because they do not want to give up their trade area to their competitors. If we combine the five destinations into one group, we get a total of 14 groups. We divide the 14 groups into 10 and 4 groups—i.e.,70 cells into 50 and 20 cells, for companies 0 and 1, respectively. The allotment is the closest to the current share ratio of 7:3. If we denote the number of groups allocated to Company 1 determined by the Ising model is *N*_g_^1^, we can use |*N*_g_^1^—*4*| as a component in the objective function of Bayesian optimization (*H*_BO_) instead of -*N*_seat_. By redistributing the delivery destinations, we can create 14 compact groups with no enclaves in each group (center and left panels in Fig. 7).

Returning to the subject of electoral redistricting, as the delivery destination optimization example above, the proposed algorithm can also allocate seats in proportion to the total (assumed) number of votes in a state. However, this seemingly fair approach of allocating seats may not be upheld by the U.S. Supreme Court with respect to the U.S. House of Representatives elections, where gerrymandering is most notable. According to Rucho v. Common Cause^29^, the US Constitution does not require proportional representation, and it cannot be said that each voter has the right to ensure that the party he/she supports wins a number of seats commensurate with its statewide support. Besides, it is impossible to find a clear and court-operable standard as to whether a given districting is excessive partisan gerrymandering or not. The judgement indicated that the U.S. Supreme Court was reluctant to intervene because gerrymandering was a nonjusticiable political question.

## Conclusion

We examined the problem of redistricting 70 cells into 14 electoral districts that consist of 5 cells, where the number of cells in which party A or B governs is equal. Besides, a cell in one district must share at least one edge with the other cells in the district (rook constraint). Consequently, enclaves are forbidden. With this assumption, a combined approach of the Ising model optimization by TS and BO maximizes the seats' biased assignment. BO adjusts the hyperparameters in the Ising model and penalizes the formation of enclaves in each district. The approach reached the theoretical upper limit without the rook constraint in 16 out of 20 trials. However, we observed that it was difficult for a party to reach the limit when the cells that the party governed had many enclaves. The essence of this algorithm that redistributing the disjointed small parts into a compact form according to a set of rules appears to have various fields of application. As such an example, we also discussed the redistribution of delivery destinations.

The problem addressed in this paper may be regarded as a kind of puzzle and be included in constraint optimization problem (COP) in the more general category. It has been proposed to solve COP as Boolean satisfiability problem (SAT)^30^. However, although SAT is a very general approach, it appears to be difficult to express all constraints of this problem in propositional logic formulas efficiently. If the related hardware and software's ability to combinatorial optimization based on the Ising model consistently proceeds, it will be possible to handle more realistic models such as larger districts and multiple votes per cell soon.

## Supplementary Information

Supplementary Information.
