# Supplementary material for: Maximizing gerrymandering through ising model optimization
Source: Sci Rep. 2021 Dec 8;11:23703. doi: 10.1038/s41598-021-03050-z (PMC8655093; doi:10.1038/s41598-021-03050-z)
Supplement: Supplementary file 1 — Supplementary Information. [file 41598_2021_3050_MOESM1_ESM.docx]

**Supplementary Information**

**Maximizing Gerrymandering through Ising Model Optimization**

**Yasuharu Okamoto**^1,2^

^1^System Platform Research Laboratories, NEC Corporation, 1753 Shimonumabe, Nakahara-ku, Kawasaki, Kanagawa 211-8666, Japan

^2^NEC-AIST Quantum Technology Cooperative Research Laboratories, 1-1-1 Umezono, Tsukuba, Ibaraki, 305-8568, Japan

**Fig. S1: Seven additional cases for gerrymandering**


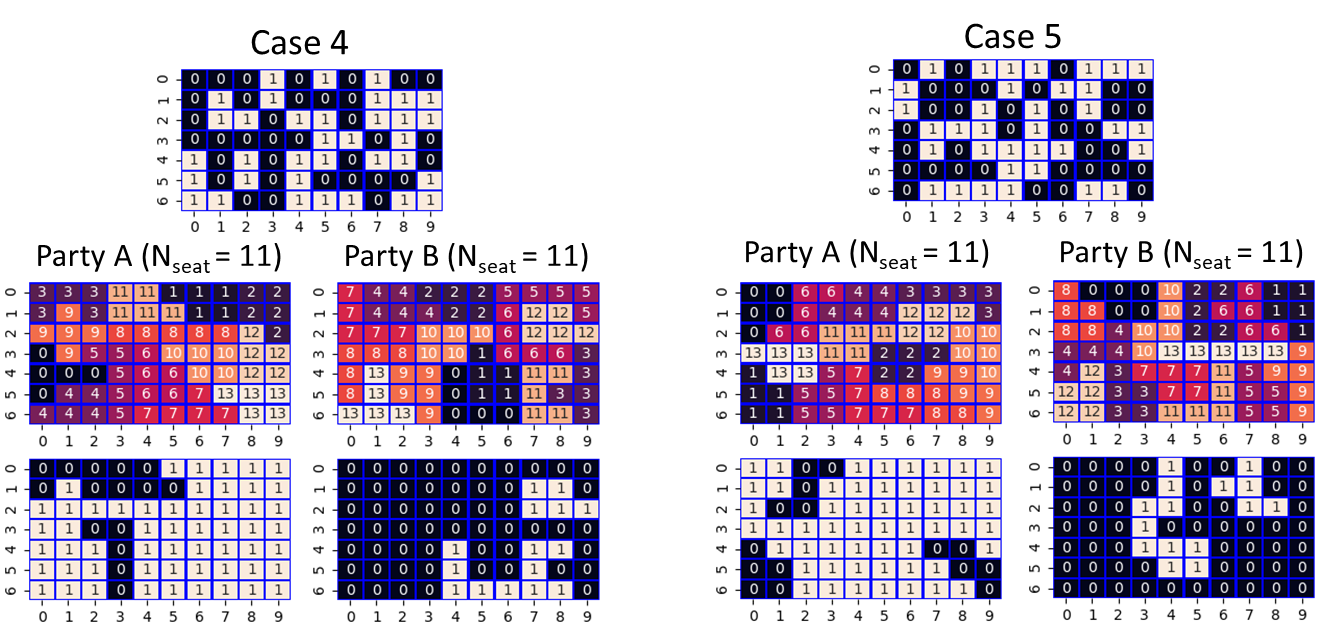


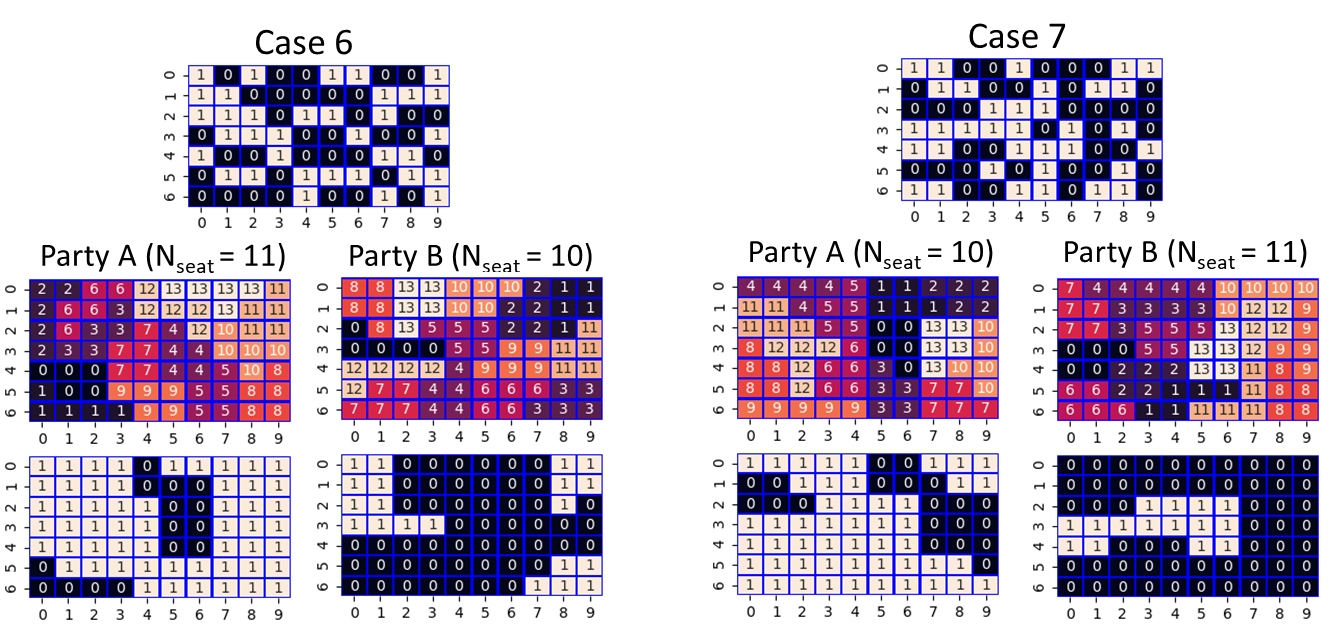


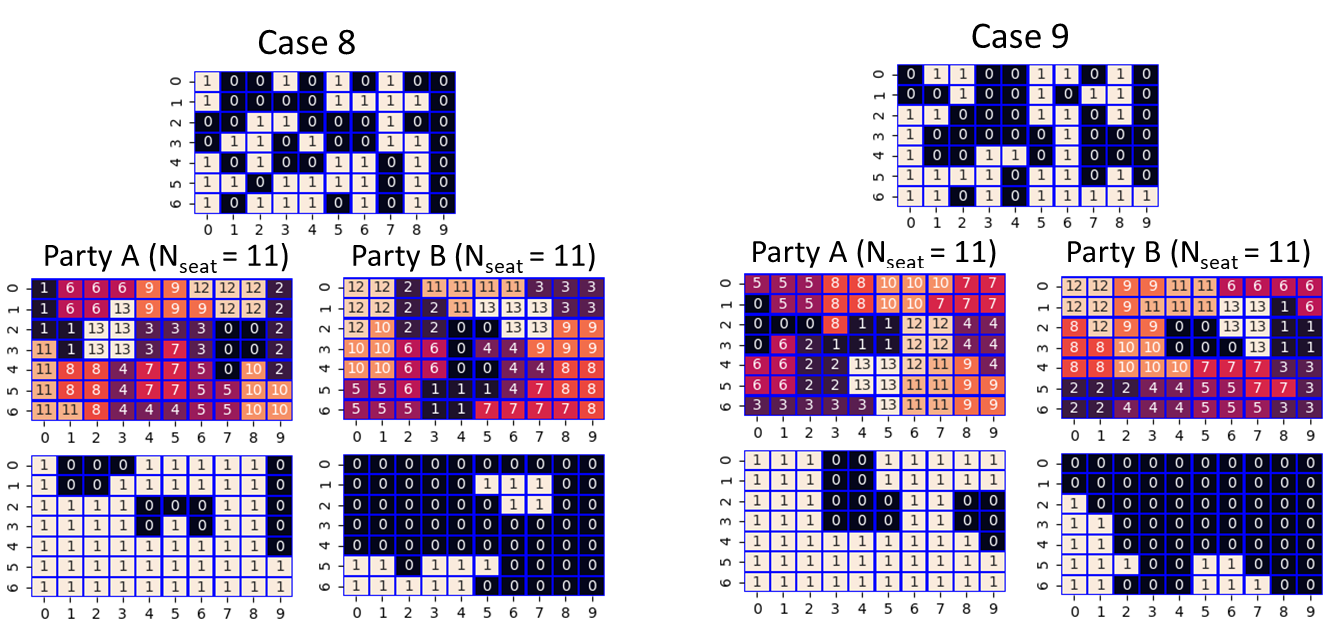


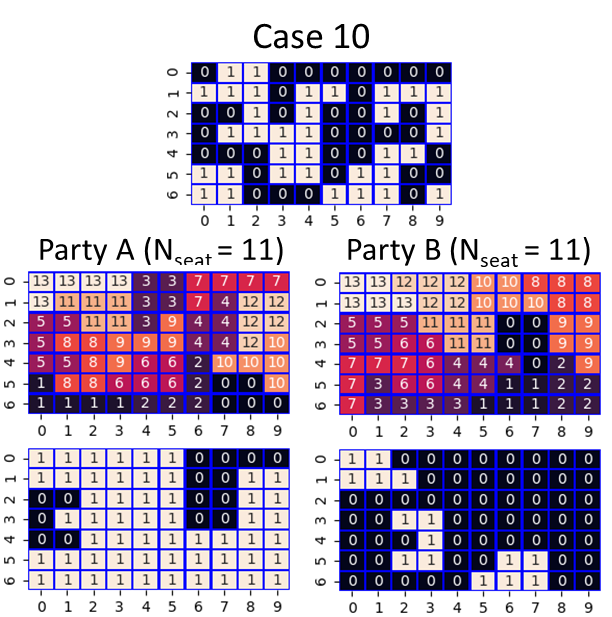


Figure S1: Other seven cases of gerrymandering with rook constraint. In each case, the top panel shows the distribution of cells where 1(0) means a cell that Party A(B) has an advantage over B(A). Middle panels show the calculated correspondence between the cells and the 14 districts indexed from 0 to 13. The bottom panels show the assignment of seats based on the calculated districting.

**Fig. S2: Queen constraint with periodic boundary conditions**


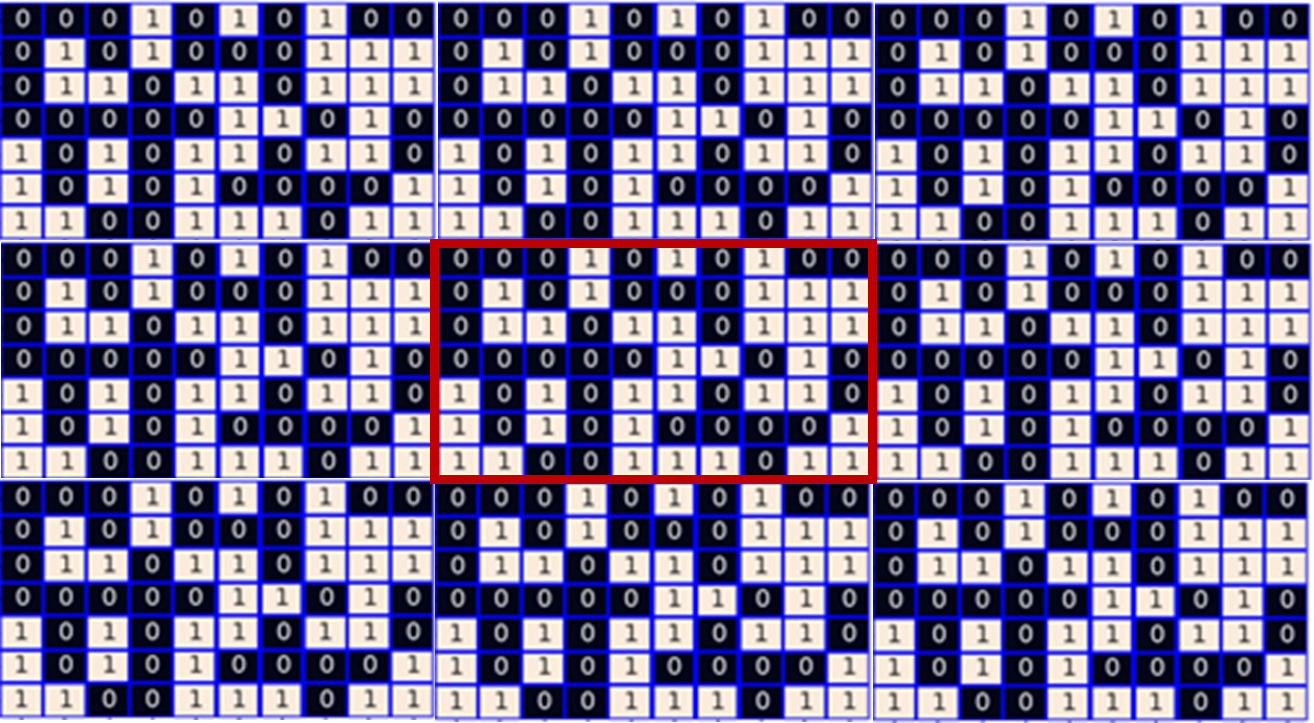


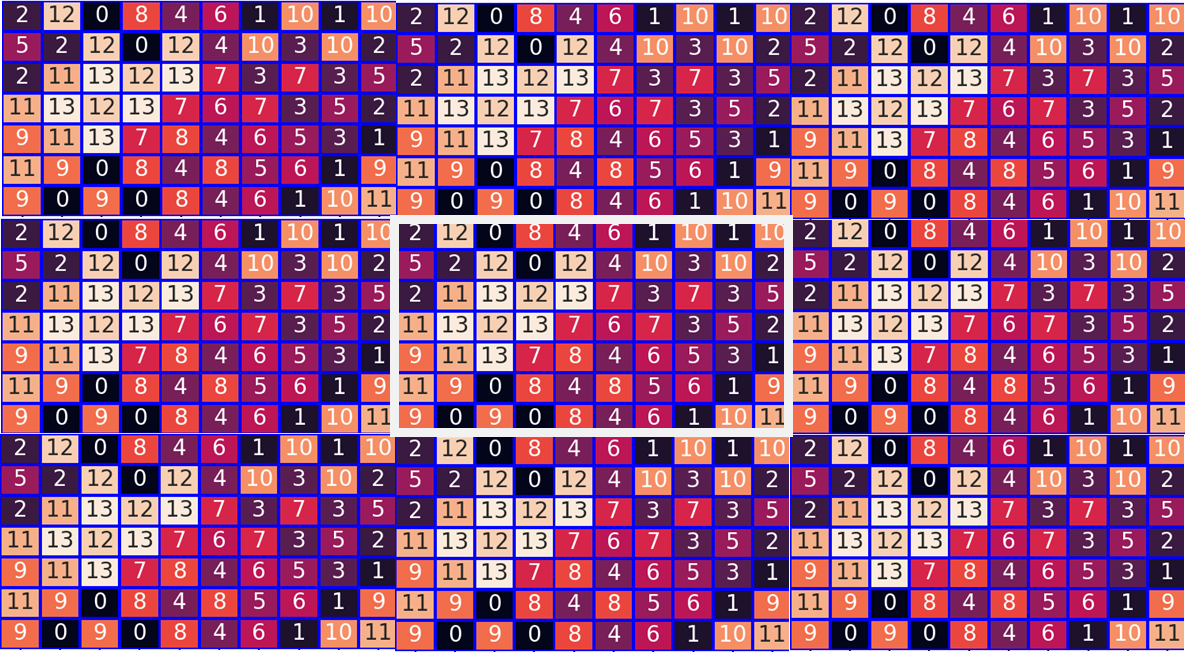


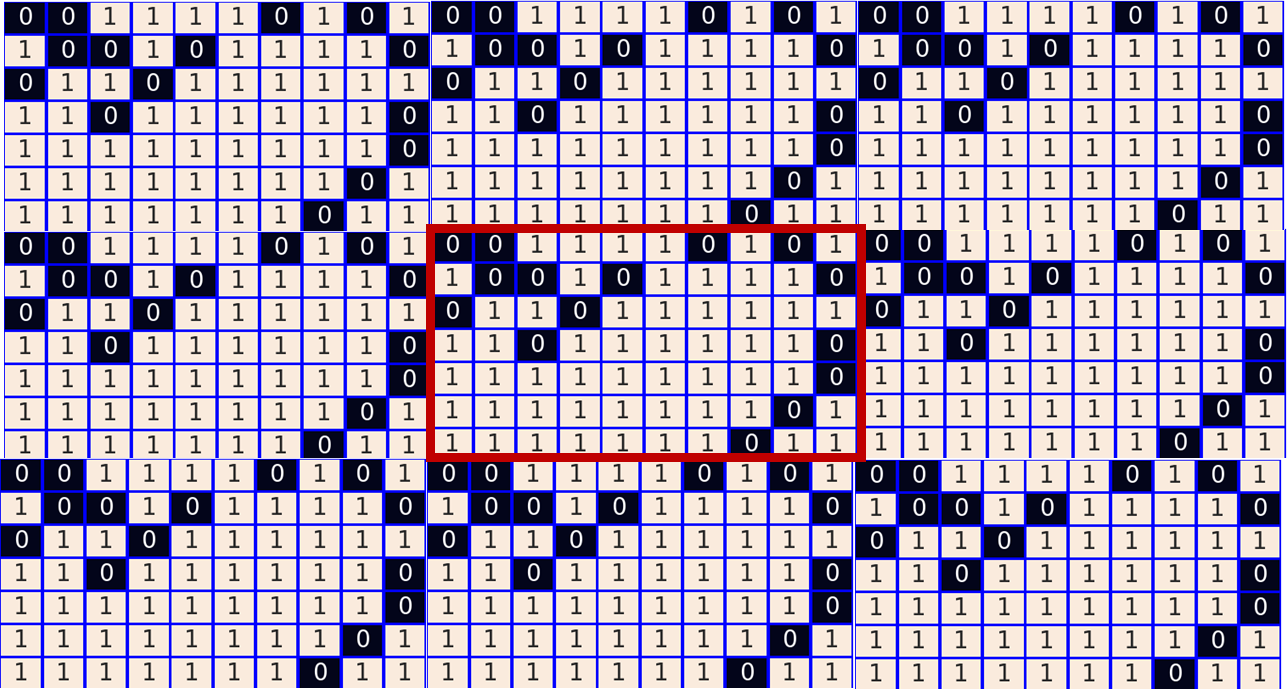


Figure S2: Basic distribution and eight images by periodic boundary conditions in gerrymandering with queen constraint. The top panel shows the distribution of the dominant party where 1(0) means a cell that Party A(B) has an advantage over B(A). Middle panel shows the calculated correspondence between the cells and the 14 districts indexed from 0 to 13. The bottom panel shows the assignment of seats based on the calculated districting.
